# Advances in OCT Angiography

**DOI:** 10.1167/tvst.14.3.6

**Published:** 2025-03-07

**Authors:** Tristan T. Hormel, David Huang, Yali Jia

**Affiliations:** 1 Casey Eye Institute, Oregon Health and Science University, Portland, OR, USA; 2 Department of Biomedical Engineering, Oregon Health and Science University, Portland, OR, USA

**Keywords:** OCT angiography, retina, retinal imaging

## Abstract

Optical coherence tomography angiography (OCTA) is a signal processing and scan acquisition approach that enables OCT devices to clearly identify vascular tissue down to the capillary scale. As originally proposed, OCTA included several important limitations, including small fields of view relative to allied imaging modalities and the presence of confounding artifacts. New approaches, including both hardware and software, are solving these problems and can now produce high-quality angiograms from tissue throughout the retina and choroid. Image analysis tools have also improved, enabling OCTA data to be quantified at high precision and used to diagnose disease using deep learning models. This review highlights these advances and trends in OCTA technology, focusing on work produced since 2020.

## Introduction

From its introduction, optical coherence tomography (OCT)^1^ had clear potential as an ophthalmic imaging modality. OCT offers volumetric imaging and can observe details similar to histology, including the identification of several different retinal tissue layers.^2^ However, unlike histology, it can be applied noninvasively to the living eye. Consequently, it is now routinely used for the detection of treatment indicators, including exudation in age-related macular degeneration^3^ and diabetic macular edema,^4^ and to monitor treatment response.^5^

Because tissue contrast in OCT is determined by reflectivity, one key limitation of the approach, as originally proposed, is difficulty visualizing capillaries and small vessels. Microvasculature has similar, though not identical, reflectance to surrounding static tissue, which means that the reflectance signal, and therefore standard structural OCT, is not ideal for identifying microvascular structure, although techniques based on artificial intelligence can help with this task.^6^ This is unfortunate because the vasculature that supplies the retina is uniquely intricate^7^ and a key component of vision processing.^8^ Also, many prevalent vision-threatening diseases include vascular pathology.^9^

OCT angiography (OCTA) is a means of supplementing structural OCT with vascular imaging. It works by trading reflectance contrast for motion contrast since blood flow can differentiate static tissue from vessels. In OCTA imaging, we refer to the motion contrast signal mainly attributable to blood flow as the flow signal, and its measurement allows the identification of vascular structure in the retina down to the capillary scale. However, because other sources of motion (e.g., ocular pulsation) also manifest as motion contrast, distinguishing the flow signal from artifacts is a major issue in OCTA. The first OCT demonstrations relied on time-domain processing.^1^^,^^10^ However, scanning rates in these systems yield imaging timescales over which sources of motion (for example, bulk motion and cardiac pulsation) extraneous to blood flow can overwhelm the flow signal. The Fourier domain approach to OCT^11^^–^^14^ eventually produced systems more than a hundred times faster than contemporary time-domain approaches, an improvement that allowed the flow signal to be isolated from other confounding sources of motion. Another important advancement was the realization that calculating motion contrast cross-section by cross-section (B-scan by B-scan)^15^^,^^16^^,^ rather than line-scan by line-scan (A-scan by A-scan),^17^ produced angiograms with higher flow detection sensitivity. One more notable development was the introduction of highly efficient flow signal generation algorithms.^18^^,^^19^ With these approaches, procedure times could be reduced to manageable lengths, and OCTA was ready for the clinic.

The first commercial OCTA devices offered fields of view confined to 3 × 3-mm field of view at high scan density, quantification consisted mostly of vessel density measurements, and images had to be manually graded. Nowadays, we are used to much larger fields of view (usually achieved by lowering scan density by some degree), several different quantifications can be accurately performed from OCTA data, and artificial intelligence algorithms can detect features with high precision. This review will describe and explain advances in OCTA, focusing on work since 2020 to demonstrate how the technology has grown from its introduction and elucidate its contemporary capabilities.

## Advances in OCTA Acquisition

The increase in scan acquisition rate provided by Fourier domain relative to time domain processing made OCTA possible, and scanning speed enhancement continues to be a driving force in OCTA technology. With faster data acquisition, developers can choose among several means to improve OCTA images. These include enlarging the field of view, improving sampling resolution, and increasing the dynamic range of flow detection. These improvements are interrelated so that once a field of view and interscan time interval are specified, sampling density is also largely determined.

Most contemporary research devices and many commercial systems use swept-source lasers (SS-OCT).^14^^,^^20^^,^^21^ These systems can typically achieve higher scan rates than alternative spectral domain (SD-OCT) systems^12^^,^^22^^–^^24^; experimental SS-OCT systems are capable of obtaining multi-MHz scan rates^25^^–^^27^ and the fastest commercial system clocks in at 400-kHz. To the best of our knowledge, the current record holder for the fastest SS-OCTA system uses a stretch-pulse active mode-locked laser and obtains a 9.4-MHz line rate, but this system was only demonstrated on rodent brain images and not the retina.^26^ SS-OCTA systems also typically use longer wavelength light sources, which benefits tissue penetration. The result is that SS-OCTA can enlarge both the transverse field of view and imaging depth for an overall larger imaging volume; however, it worth noting that the price of these systems remains relatively high due to the swept source laser component.

### Wide-Field OCTA

Restricted fields of view were an important limitation in the first OCTA devices. With a given scan rate, instrument design must balance the triple bind of procedure time, field of view, and sampling density. Early wide-field OCTA studies used montage, usually including several OCTA scans at different locations.^28^^–^^34^ This approach is okay for research but cannot be easily adapted to clinical practice.

**Figure 1.**
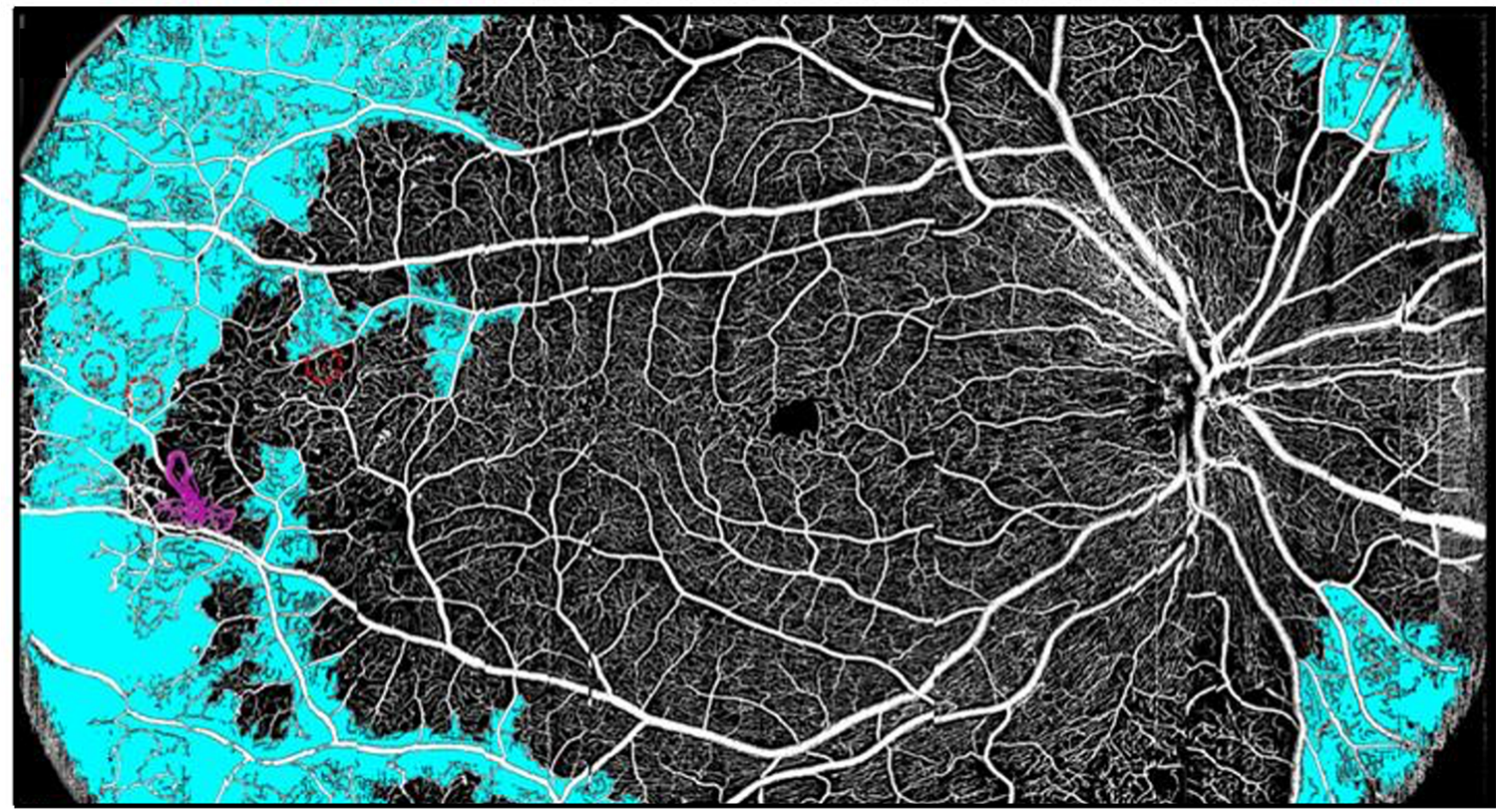
A single-shot OCTA en face image of an eye with DR across a 23 × 12 mm field of view, using a wide-field OCTA system developed by Wei et al.^31^ This system incorporates a self-tracking method to remove disrupting motion artifacts,^32^ and OCTA processing was performed using phase-stabilized complex decorrelation, an approach that efficiently achieves a high flow detection sensitivity.^33^ Pathologic features include non-perfusion area (*teal*), retinal neovascularization (*magenta vessels*), and microaneurysms (*red, with dotted circles*). Most of the pathology in this image is located outside of a conventional central macular field of view, demonstrating the usefulness of wide-field imaging in this disease. Reproduced with permission from Hormel et al.^34^

Improved scanning speeds from SS-OCTA offer a way to extend the field of view without impractical procedure times. For example, an SS-OCTA system equipped with a 1.68-MHz Fourier-domain mode-locked laser can achieve a 23 × 17-mm field of view with Montage.^35^ Jia's group developed a prototype 400-kHz SS-OCTA system (short cavity swept-source laser; Axsun Technologies, Billerica, MA, USA), which demonstrated a single-shot 23 × 12-mm field of view^31^ (Fig. 1) in retinal clinical studies. It has been recently upgraded to a 500-kHz SS-OCTA system (vertical cavity surface-emitting laser; Thorlabs, Newton, NJ, USA) with improved sensitivity, generating wide-field OCTA in both superficial and deep slabs.^34^^,^^36^ Single-shot systems can reduce procedure complexity and are also useful for patients who may have difficulty with fixation. These properties are useful for clinical practice, and commercial systems can now also perform wide-field single-shot imaging (15 × 15-mm for Zeiss PLEX Elite [Zeiss, Oberkochen, Germany], 26 × 21-mm for Intalight DREAM [Intalight, San Jose, CA, USA]) in something like two minutes including acquisition and processing (depending on the patients ability to fixate and other external factors), but they achieve this by downsampling.

**Figure 2.**
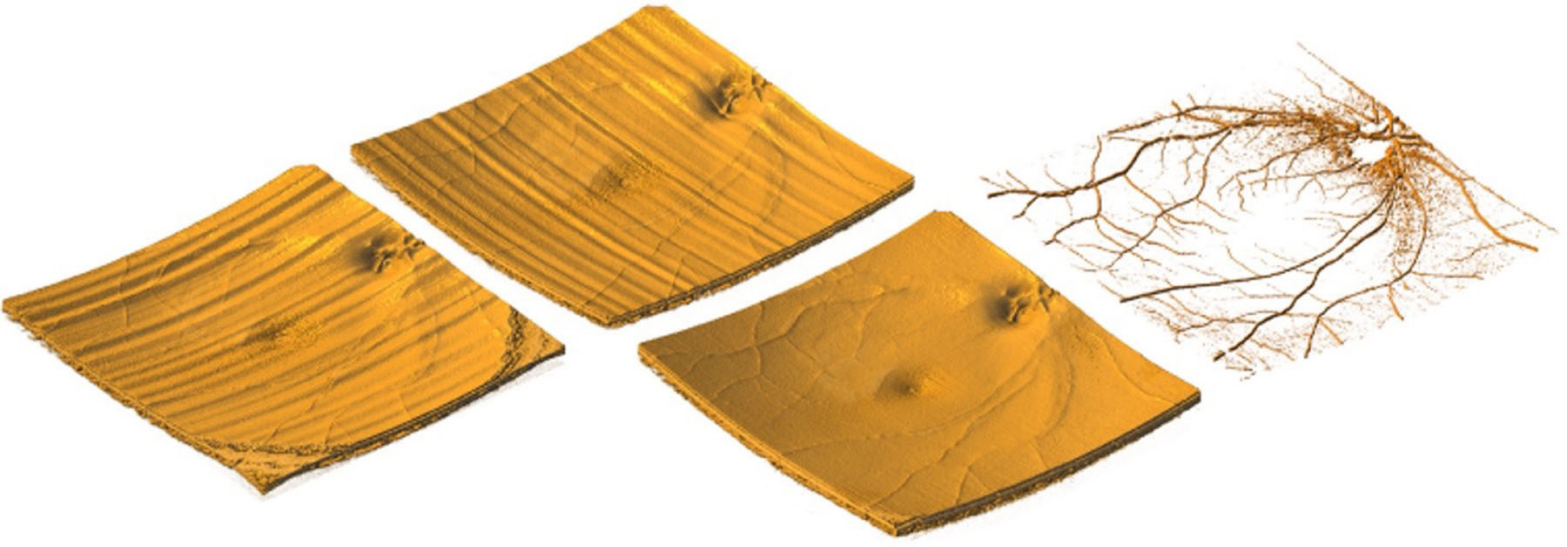
Volumetric motion correction using orthogonal scanning. Left to right: X-fast, Y-fast, merged OCT, and merged OCTA volumes at equal transverse and axial resolution. With permission from Ploner et al.^43^

Capturing high-quality wide-field images is not as simple as just incorporating a high-speed swept source laser into a device. One issue is that as fields of view increase distortions due to mapping a curved surface to a flat display (like a computer screen) become more severe; these can lead to significant differences in feature quantification and should be corrected.^37^ A second is ensuring that the device sample arm covers a sufficiently large range to image the eye at different depths, which also becomes more difficult in widefield imaging. Another problem exacerbated by wide-field imaging is unwanted eye motion (for example due to ocular pulsation, microsaccade, drift, or blinking) and is a particularly acute issue for OCTA because the technique is based on using motion contrast to identify vessels. Because wider fields of view often require longer procedures times motion due to sources other than blood flow can become more severe. Many motion correction approaches rely on allied imaging modalities such as scanning laser ophthalmoscopy and others, examples being Vienola et al.^38^^–^^40^ These approaches are examples of active tracking and complicate imaging systems by requiring additional hardware. As an alternative, contemporary systems can rely on real-time OCTA processing for motion correction^41^ or use passive tracking in which problematic regions are re-scanned by the device.^32^ These approaches are aided by the use of efficient and sensitive OCTA processing algorithms, for example phase-stabilized complex decorrelation OCT angiography.^33^ Registering across orthogonal scanning (as, for example, in Optovue devices [Optovue, Fremont, CA, USA]) directions is another way to address motion artifacts volumetrically (Fig. 2).^42^^,^^43^

There are obvious reasons for seeking to enlarge the field of view in OCTA imaging. To begin with, important pathology can be located in the periphery. Examples are lesions in retinopathy of prematurity^44^ and retinal neovascularization (RNV) in DR.^36^ Wide-field OCTA is an advantageous technology for RNV because it can identify neovascularization that is not noticeable in clinical examination,^45^^–^^47^ and in head-to-head comparisons, wide-field OCTA shows comparable performance to wide-field fluorescein angiography and superior performance to color fundus photography for RNV detection.^48^ Even when pathology can be identified in conventional fields of view, wide-field imaging may be necessary to reveal its full extent. An example is ischemia caused by branch retinal vein occlusion^34^ or non-perfusion area in diabetic retinopathy (DR).^49^^,^^50^ And finally, we know from other imaging modalities and gene expression profiling that the posterior pole and peripheral retina have different physiological characteristics.^51^

### High-Resolution OCTA

**Figure 3.**
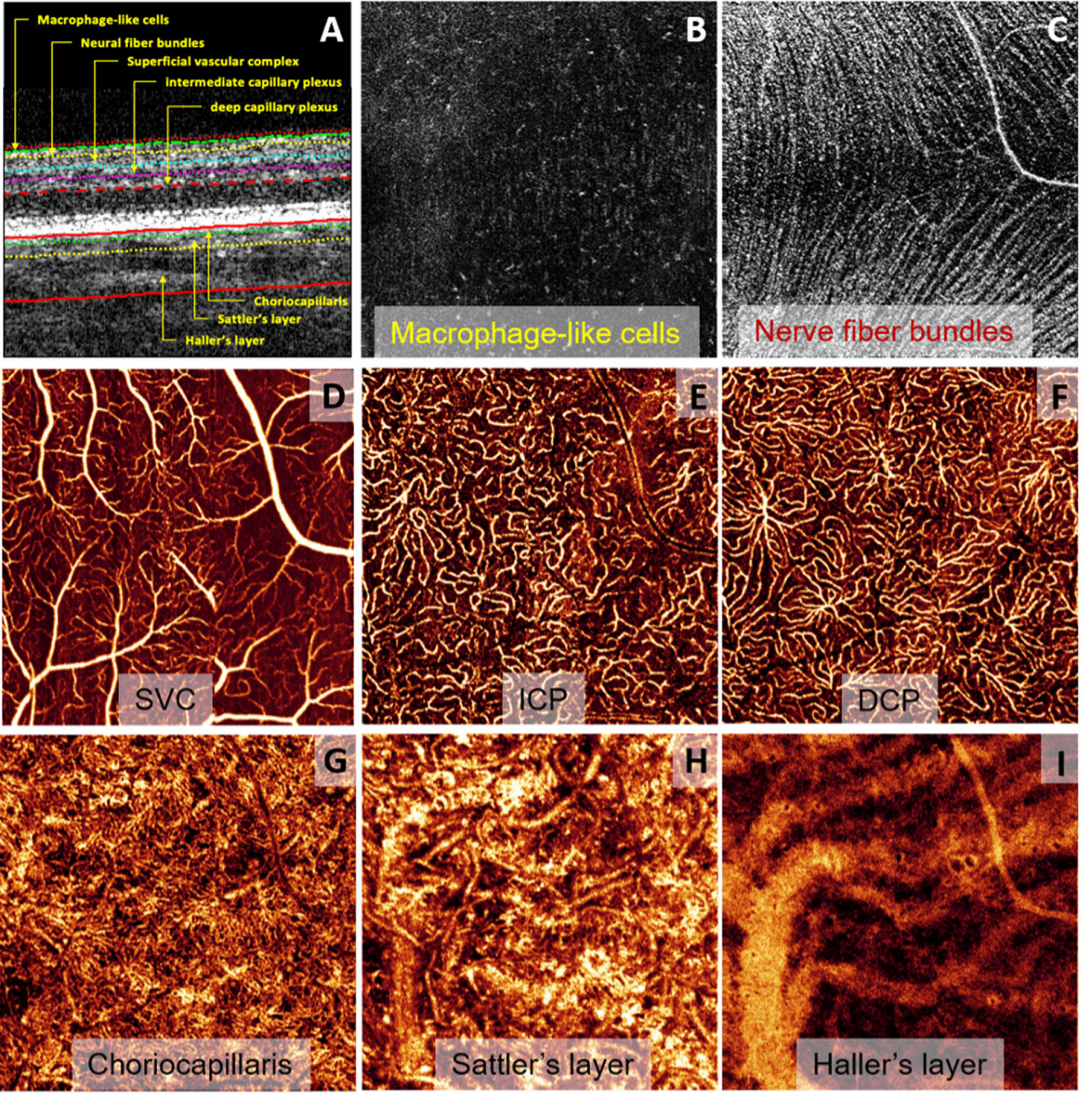
High transverse resolution structural OCT and OCTA images of retinal tissue from the vitreous to Haller's layer. (**A**) Cross-sectional image showing the boundaries of the tissue layers and plexuses imaged in the following en face images. (**B**) Macrophage-like cells visualized in the structural channel above the inner limiting membrane. (**C**) Nerve fiber bundles in the nerve fiber layer. (**D**–**F**) Vessels in the retinal plexuses display distinct morphology. (**G**–**I**) Choroidal vessels similarly display unique characteristics when imaged at high resolution. With permission from Hormel et al.^34^

Transverse and axial resolution in OCTA are decoupled and can be separately optimized. Axial resolution can be important in structural OCT to improve the precision of thickness measurements^52^^–^^56^ but in OCTA we often use en face data representations because the retinal plexuses and choriocapillaris are each laminar tissues oriented largely perpendicular to the scan direction.^57^^–^^59^ Such visualizations are created by projecting over specific tissue layers. For these displays it is important to maintain transverse resolution in both superficial and deep tissue to best capture vascular morphology (Fig. 3).

Transverse resolution can be limited by the anatomy of the eye. Pupil size imposes an upper limit on beam diameter, and, hence, diffraction limited transverse resolution. However, most systems eschew this limit because wider beam diameters are more vulnerable to optical aberrations in the field outside of the central macula. A common choice for OCT has been a 2-mm beam diameter, which gives ∼15-µm transverse resolution. This is actually too low to resolve capillaries at their anatomic width (which can be as small as ∼5 µm^60^) and is one reason some researchers prefer to skeletonize vessels when calculating vessel density. However, the challenges from wider beam size can be alleviated by real-time OCTA quality display^41^ and high sampling density.^31^

Aberrations can also be corrected in hardware. The conventional way is with adaptive optics (AO).^61^ Early AO-OCTA systems used AO optical setups that included deformable mirrors and wavefront sensors.^62^^,^^63^ The increased cost of these components makes such systems difficult to justify as a translational technology. Sensorless AO-OCTA systems have been demonstrated and provide similar image quality but could provide a better option for clinical adoption^64^ or other applications such as murine imaging.^65^ It is, however, noteworthy that a similar sensorless AO-OCT system and other AO-OCT devices can observe capillaries without using motion contrast in anterior plexuses; that is, without OCTA processing.^66^ This is more difficult in the superficial vascular plexus and in particular, for radial peripapillary capillaries, which have very similar reflectivity to the surrounding static tissue.^67^ A purely reflectance signal-based approach also loses the functional information from OCTA- abnormal flow in vessels is not obvious. Another benefit of the high-resolution imaging in these system is that they can avoid projection artifacts, which are a major complication in OCTA imaging that will be discussed below (Section: Artifact Correction).

### High Dynamic Range OCTA

Flow signal magnitude in OCTA depends on both intrinsic features of the tissue being imaged—which include erythrocyte density, vessel caliber, and flow velocity—and extrinsic factors, the most important of which is interscan time. Even keeping other intrinsic factors and interscan time fixed, flow signal magnitude is only linear with flow velocity within a limited flow velocity regime outside of which flow is either undetectable (too slow) or saturated (further increase in flow velocity does not increase flow magnitude).^68^ In practice what this means is that there isn't a single ideal interscan time because different flow velocity regimes are better imaged by different interscan times.

High-dynamic range OCTA (HDR-OCTA) recognizes this problem and solves it by processing several repeat scans which can be combined to sample at multiple interscan times.^69^ Of course, many devices capture more than two scans to construct the OCTA signal. But most simply average the signal across a single interscan time interval Δ*t* to enhance it. Instead, HDR-OCTA also measures the flow signal between multiples of the interscan time (e.g., 2Δ*t*, 3Δ*t*, etc.) to extend the flow detection regime to slower flow. With enough repeats Δ*t* can also small enough to extend the dynamic range in the opposite direction. An efficient way to implement this approach is by using efficient scanning patterns. OCTA systems usually use raster scanning protocols. But raster scanning is actually inefficient because it wastes time on flyback, during which the system realigns at the edge of a sampling region without collecting data. Bidirectional and interleaved scanning are alternatives that both eliminate flyback time.^70^^,^^71^

**Figure 4.**
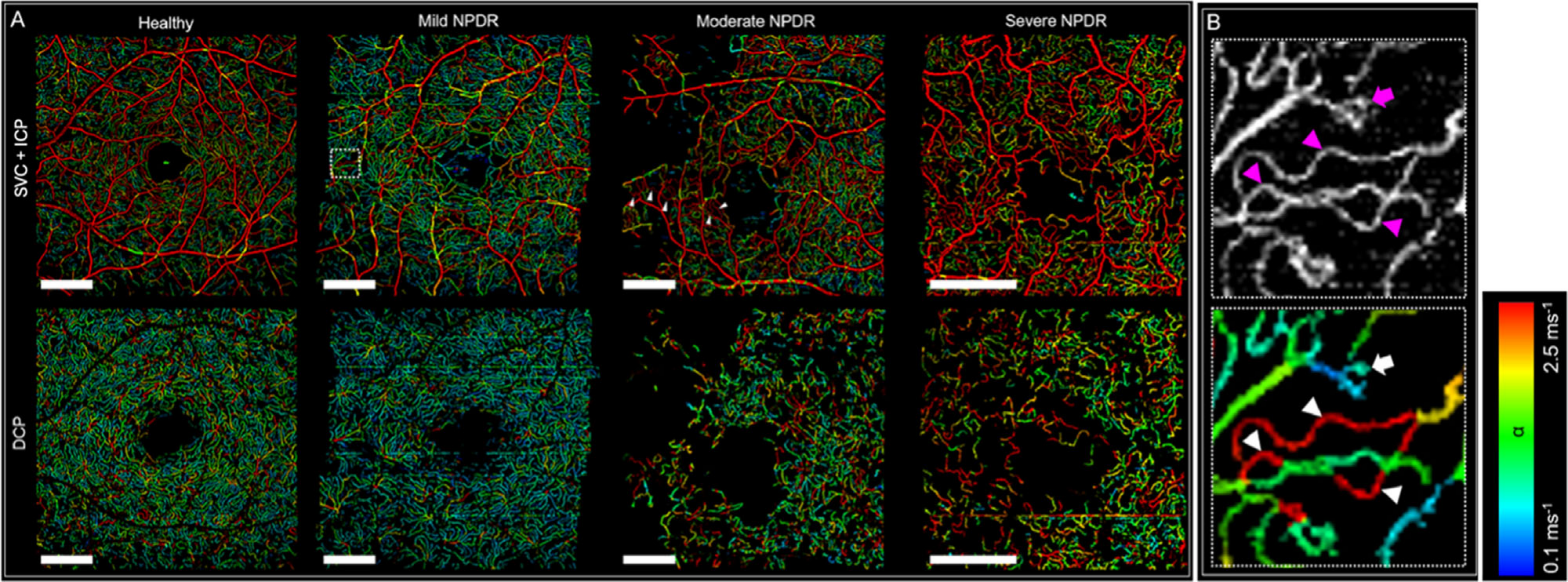
Flow speed measurement using variable interscan time analysis (VISTA). (**A**) VISTA images showing the combined superficial vascular complex and intermediate capillary plexus (*top row*) and the deep capillary plexus (DCP) of volunteers with different severities of DR. Flow speeds α are indicated by color (*panel on right*). (**B**) Blow-up of the area within the *dotted rectangle* in (**A**), showing tortuous capillaries with high α (*triangles*) and focal bulge with low α (*arrow*). With permission from Hwang et al.^74^

Actually, using multiple interscan times can do more than just extend the dynamic range. By comparing flow signal magnitude across multiple interscan times flow speed can also be assessed; this method is called variable interscan time analysis.^72^^,^^73^ Beyond sampling flow at different intervals the multiple interscan times are important for correcting for pulsatility (Fig. 4).^74^ Reliable measurement of flow speed can be an important improvement to OCTA imaging since it extends the functional aspect of the technique from an all-or-nothing, flow-or-not visualization to assess pathologically slow/fast flow could be detected.

### Handheld OCTA

**Figure 5.**
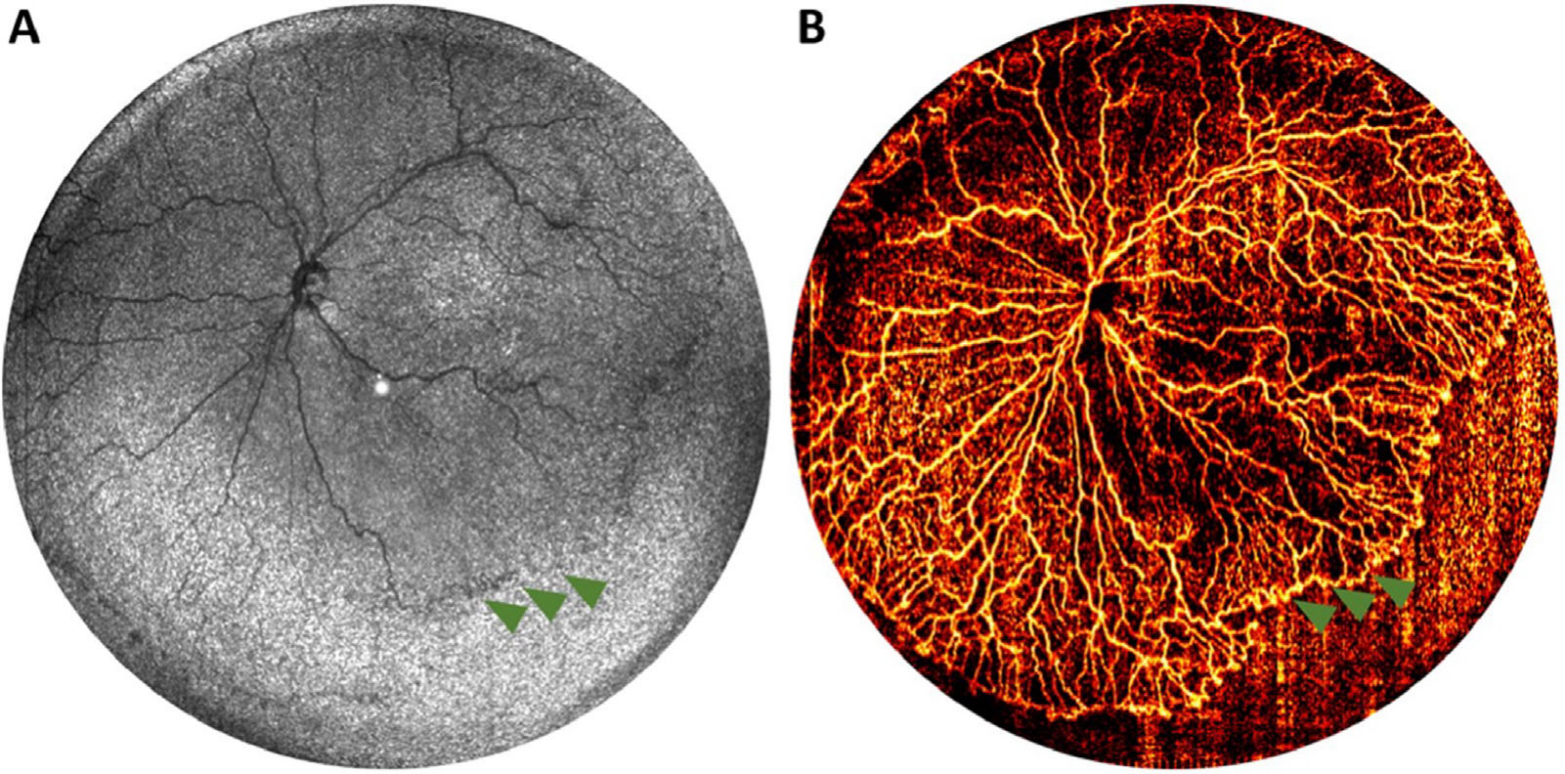
A 140° field of view OCTA images acquired with a handheld device. (**A**) En face OCT image of the right eye from an infant (born at 23 weeks gestation, 593 grams, and imaged at 38 weeks postmenstrual age) with retinopathy of prematurity stage 1, where the fibrovascular ridge is marked by *green arrowheads* but is not distinctly visible in the structural en face image. (**B**) The corresponding en face OCTA heatmap, which enhances the visualization of the vascularized retina, providing greater clarity and detail. With permission from Ni et al.^75^

Pediatric populations provide a different context for OCTA imaging. With infants we cannot use fixation targets or otherwise rely on a cooperative patient, and so we use handheld devices. These devices must balance different design considerations than conventional OCTA devices, including a short working distance and limited procedure times (because babies will begin to cry). The lower limit for working distance is a contact probe, but this should not necessarily be regarded as a limitation for OCTA devices.^75^ But shorter procedure times do represent an improvement for the technology. Since the first demonstration of OCTA in a handheld system,^75^ the time required to image similar fields of view has dropped; OCTA requiring 3.2 seconds for a 36° has been demonstrated in a handheld device.^76^ Swift image acquisition can help reduce motion artifacts, but these can also be addressed separately.^77^ Handheld devices also face a unique requirement for ergonomics; for example, OCTA devices specialized for supine imaging have been demonstrated.^78^ Finally, similar to conventional OCTA, field of view in handheld devices is increasing. The current state of the art for handheld OCTA is a 140° contact probe system that uses an 800-kHz swept source laser for sampling and a telecentric lens relay system to reduce beam wandering (Fig. 5).^79^

## Advances in OCTA Image Processing and Analysis

In the preceding we discussed improvements in the type and quality of data that OCTA can acquire. Now we want to discuss advances in what we can do with that data.

### Artifact Removal

Both visualization and quantification of OCTA data can be improved by addressing artifacts. Unfortunately in OCTA extra care must be taken to correct artifacts relative to structural OCT.

Projection artifacts are a type of artifact unique to OCTA.^79^ They duplicate superficial vascular patterns in deeper tissue layers. Visually they disrupt interpretation of vascular morphology in these deeper layers, for example by obscuring the lobular pattern of deep capillary plexus vasculature. On cross-section they create long tails on vessels. Quantitatively they lead to measurements of vascular parameters that should be isolated to specific plexuses to instead be smeared across the target region and superficial flow patterns. Furthermore, they can disrupt detection of macular neovascularization (MNV).^80^

**Figure 6.**
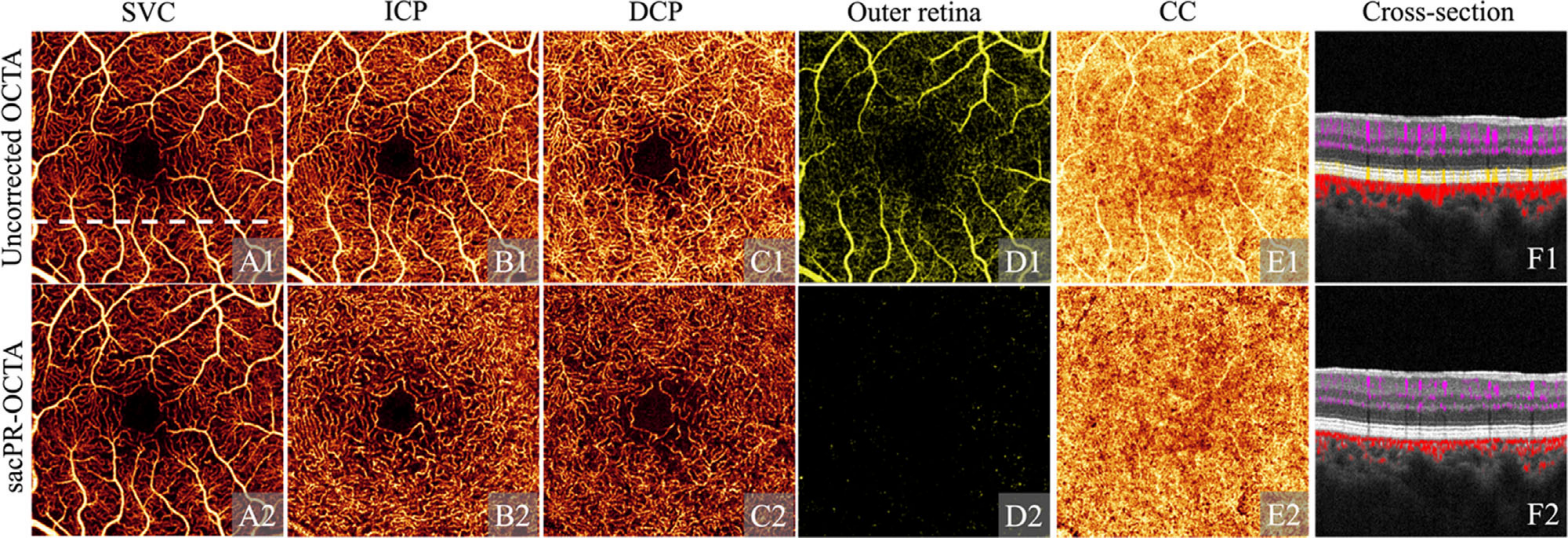
A normal eye imaged with uncorrected OCTA (row 1), projection-resolved signal attenuation compensated OCTA (sacPR-OCTA, row 2) showing en face OCT angiogram of the superficial vascular plexus (SVC, column A), the intermediate capillary plexus (ICP, column B), the deep capillary plexus (DCP, column C), the outer retina (column D) and the choriocapillaris (CC, column E), and the cross-sectional structural OCT overlaid with color-coded flow signal (column F; *violet*: inner retina; *yellow*: outer retina; *red*: choroid) at the position indicated by the white dotted line in A1. The sacPR-OCTA algorithm removed more artifacts while maintaining vascular integrity, and eliminated artifactual vessel tails (F). With permission from Wang et al.^81^

Projection artifacts are caused by time-varying shadowing because of flow in overlying vessels and photons being scattered by multiple scatting events. They attenuate with distance from the source vessel. State-of-the-art projection artifact removal can account for this effect to remove artifactual flow signal volumetrically without over-processing removing in situ flow (Fig. 6).^81^ This contrasts with early methods for projection artifact removal that both over-processed images of deeper tissue and worked only for en face images rather than volumetrically.^82^^–^^84^

Another way that image quality can be improved is through noise reduction. Classical approaches to this topic typically consist of applying handcrafted filters to images in order to augment specific features. For example, vesselness filters can be applied to en face OCTA angiograms to emphasize vascular patterns.^85^ Alternatively, background can also be reduced. In OCTA bulk motion is a large contributor to background, so in this case an example is an iterative approach to reducing its signal strength.^86^^–^^88^ Statistical approaches that attempt to estimate flow signal value are also effective and can be applied to deeper layers where signal attenuation is a factor.^89^

**Figure 7.**
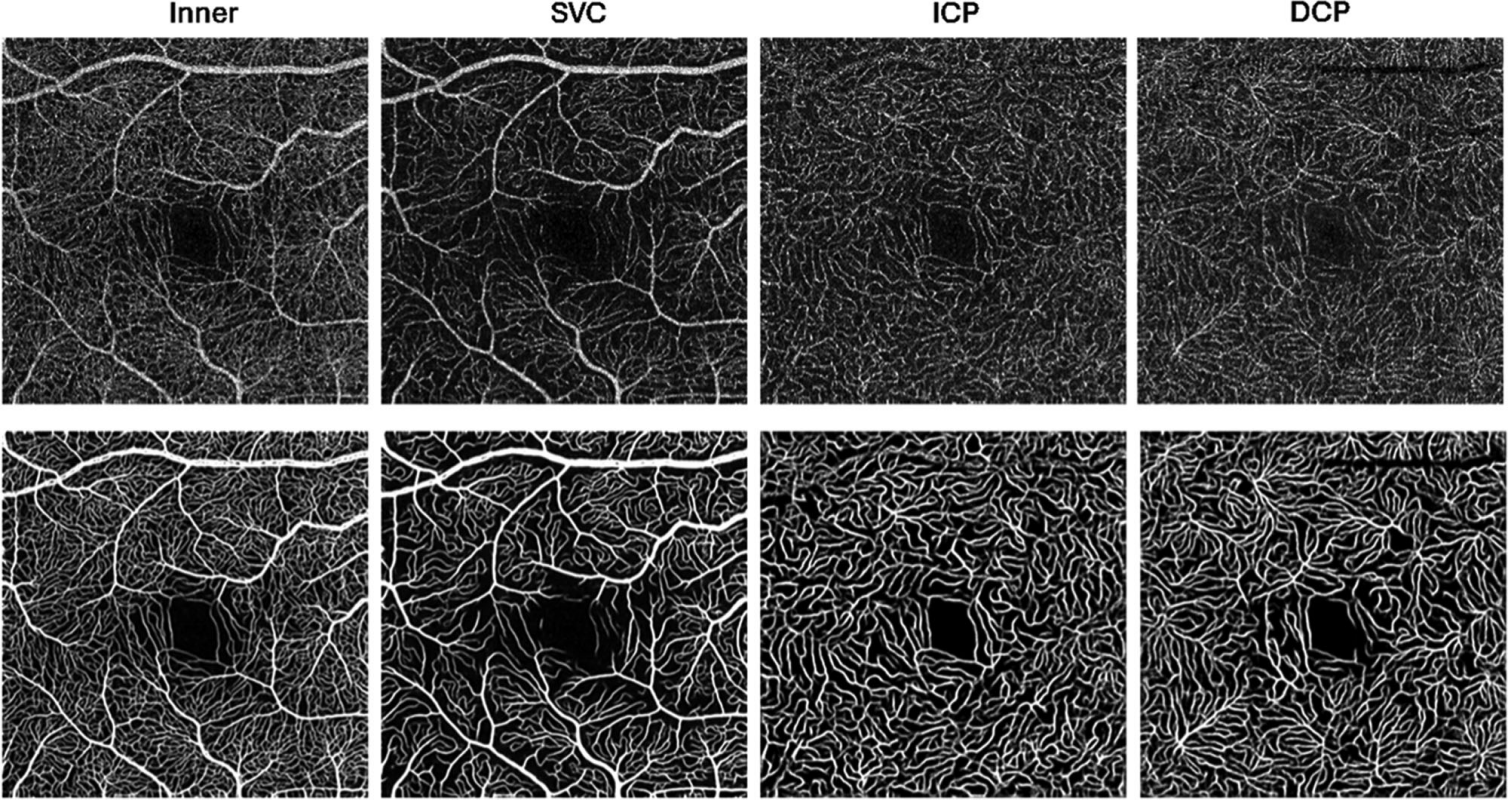
Artifact-free OCTA image enhancement using deep learning. Top row: raw images; bottom row: images denoised with a deep learning network. Note that this method did not produce artifactual vessels, as can occur with handcrafted vesselness filters. With permission from Gao et al.^90^

A disadvantage to handcrafted algorithms like these is that they lack context awareness that can be trained into neural networks. This is a clear advantage for deep learning-based approaches. For denoising the subtlety is in choosing denoised images: how do we select cleaner images for networks to learn from? One option is train networks to reconstruct high-definition scans from low definition, undersampled scans; interestingly, networks trained in this way were actually able to improve scan quality in high-definition scans, as well as undersampled ones (Fig. 7).^90^^,^^91^ Another approach is to train networks to improve angiograms constructed with a lower number of repeats from angiograms with more repeats, because the higher number of repeats improves signal quality.^35^^,^^92^

### Deep Learning in Feature Detection and Segmentation

**Figure 8.**
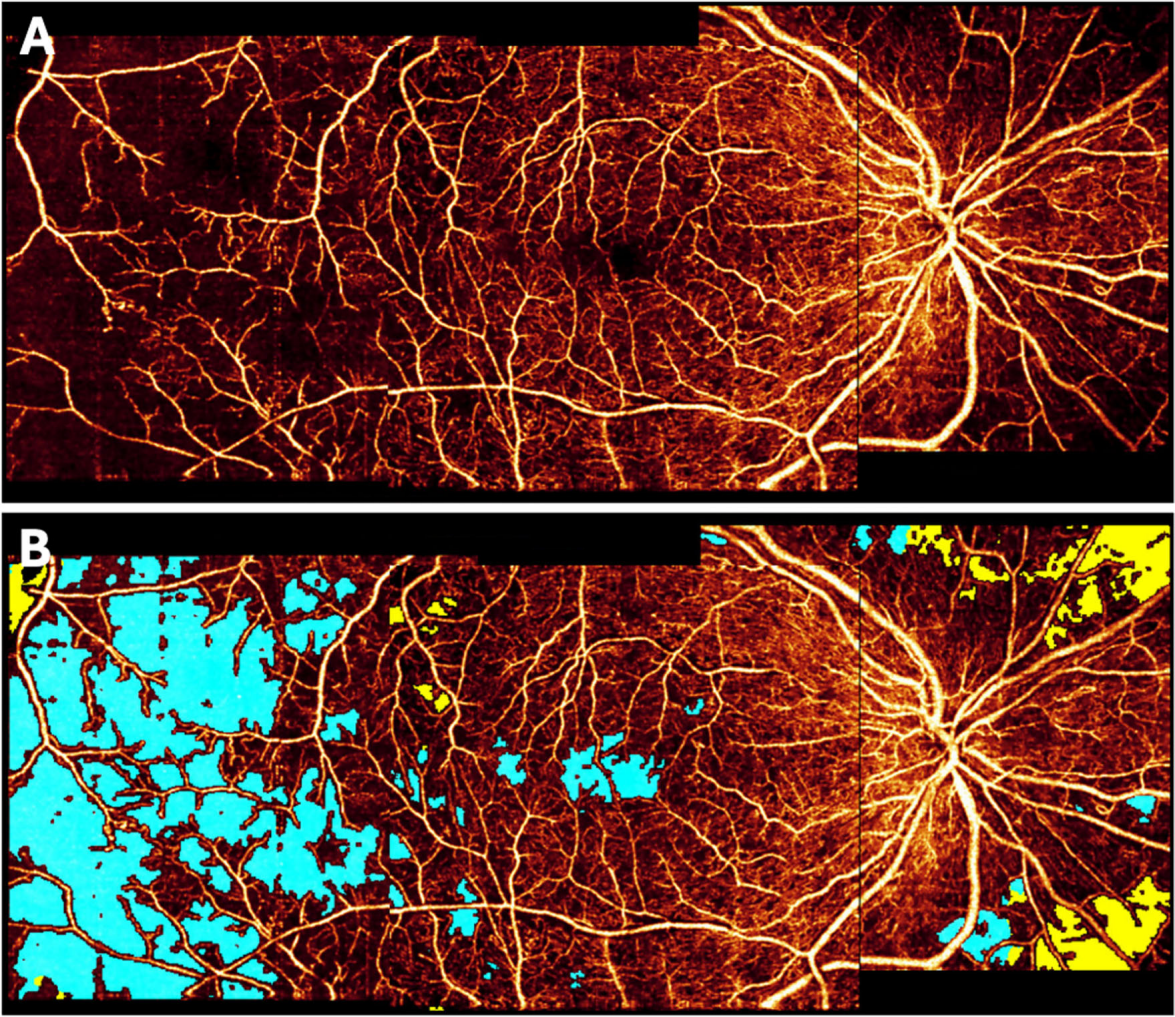
Automated NPA segmentation results on montaged wide-field OCT angiography imaging for an eye with severe nonproliferative DR. (**A**) Angiogram image; (**B**) angiogram with labeled NPA (*teal*) and shadow artifacts (*yellow*). By avoiding detecting shadow as NPA, this deep learning model improves NPA quantification. With permission from Guo et al.^104^

Quantification of OCTA angiograms can stage and diagnose prevalent vision threatening diseases.^93^^,^^94^ Originally, OCTA quantification focused on vessel density^95^^–^^99^ or vessel morphology metrics such as tortuosity or fractal dimension.^100^ These approaches have been reviewed elsewhere.^80^^,^^93^ An alternative proxy to vessel density for perfusion loss is non-perfusion area (NPA), which is defined as vessel-free regions exceeding an area threshold.^101^^,^^102^ An advantage for NPA over vessel density is that it can provide a visual cue for the location of perfusion loss, which can be useful for assessing disease processes (Fig. 8). With the aid of deep learning NPA can be quantified in separately in individual plexuses^103^ and wide-field images.^104^ Such measurements correlate with retinopathy^105^ and glaucoma severity.^106^^–^^110^

Vasculature in the posterior segment is perhaps the most intricate in the human body. As such, not all measurements are suitable in every anatomic region. Although NPA is useful for assessing perfusion in the retinal plexuses, it is not well suited for assessing perfusion defects in the choriocapillaris where vasculature is dense, and perfusion can be lost without correspondingly large vessel-free regions. Instead, perfusion loss in the choriocapillaris is best assessed by detecting flow deficits. This task is difficult because vessel density in the choriocapillaris is high enough that individual capillaries are difficult to resolve, and, because of its location posterior to the retina, requires imaging in a region necessarily affected by signal attenuation and possibly complicated by overlying pathology such as drusen or retinal fluid.^111^ But if it is difficult to measure flow deficits, it is also useful—flow deficit quantification can predict which late-stage form of age-related macular degeneration is likely to develop^112^ and predict how rapidly the hallmark features for these stages (macular neovascularization and geographic atrophy) will develop^113^^–^^116^ and where.^117^ Flow deficits also correlate with disease severity in DR.^118^

**Figure 9.**
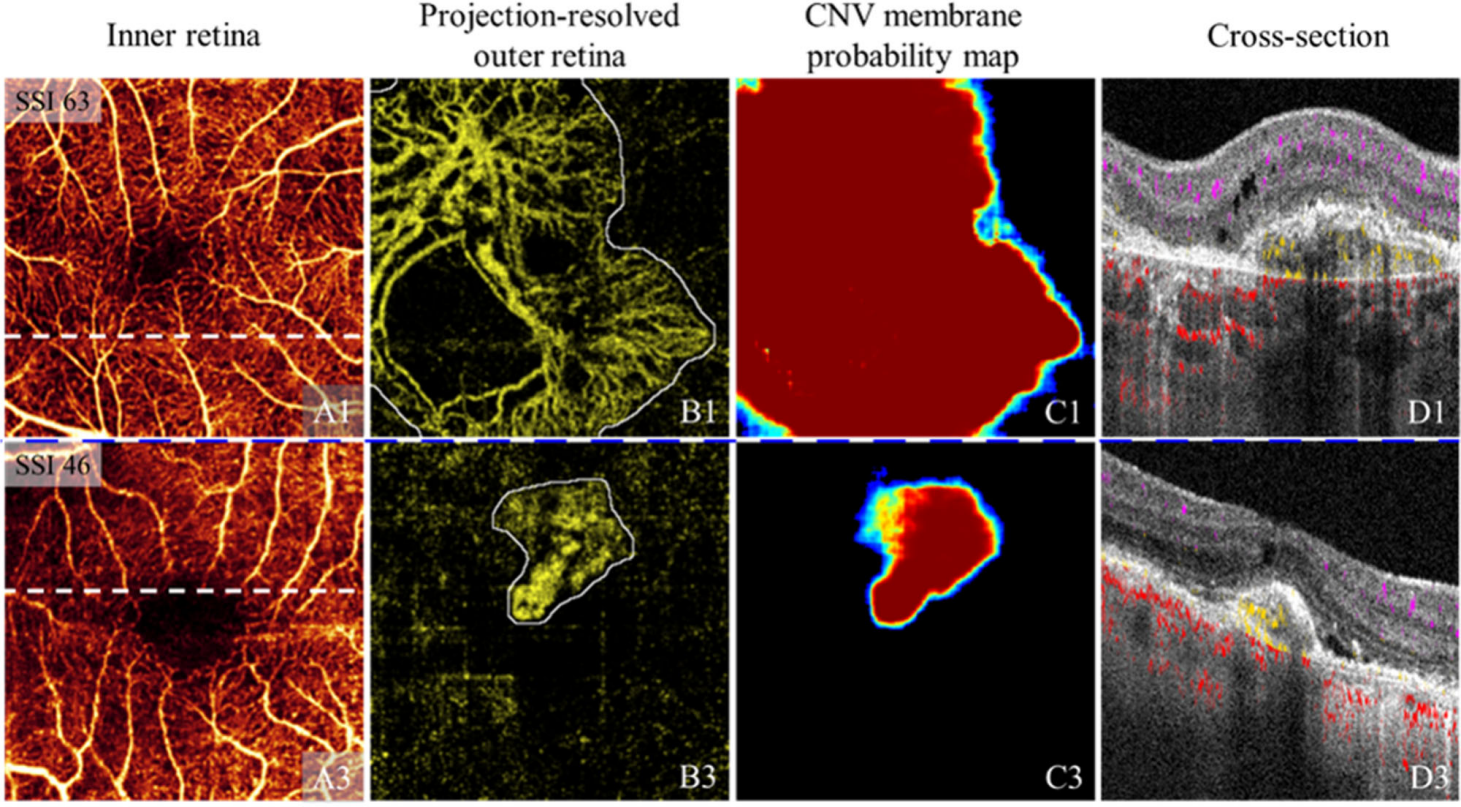
MNV membrane segmentation on 3 × 3-mm scans. (**A**) Inner retinal angiogram. (**B**) Projection-resolved outer retinal angiogram with MNV membrane ground truth (*white outline*). (**C**) MNV probability map. (D) Structural OCT cross section at the location of the white line in (**A**), with flow signal overlaid (*violet*: inner retinal; *yellow*: pathologic outer retinal; *red*: choroidal). Case 1 shows a large MNV lesion that cannot be fully imaged using a small scanning window. Case 2 shows a small MNV lesion in a defocused scan with strong residual artifacts. These cases illustrate the versatility of an deep learning based approach for MNV segmentation. With permission from Wang et al.^121^

MNV is itself an excellent target for OCTA, but visualization is complicated by the posterior location of the pathology. It is best analyzed with projection artifact removal.^81^^,^^119^ With projection artifact removal OCTA is a powerful tool for interrogating MNV. As a volumetric imaging modality OCTA can distinguish MNV type,^94^ and artificial intelligence can be used automatically to detect and segment lesions (Fig. 9),^120^^,^^121^ enabling efficient monitoring of lesion development and treatment response.^122^^–^^124^

Both MNV and NPA represent features with mature image analytic approaches already developed for segmentation and detection. There are other features OCTA can address. Microaneurysms can be detected and categorized by appearance, transverse and axial location, and the presence/amount of flow signal using OCTA.^125^^–^^130^ The last two capabilities stand in contrast to dye-based angiography, which remains useful for detecting microaneurysms because it can image the periphery.^131^^,^^132^ (Although as discussed above contemporary OCTA devices are incorporating larger fields of view.)^34^^–^^36^ Another pathology that OCTA is excellent for identifying that benefits from larger fields of view is retinal neovascularization. OCTA is superior for retinal neovascularization detection in comparison to dye-based angiography because it can readily identify neovascular vessels due to their location above the internal limiting membrane.^46^^,^^133^ But similar to microaneurysms, retinal neovascularization detection should really be performed on wide-field systems to avoid missing peripheral pathology.^45^^,^^48^ Other examples of pathology in which OCTA provides superior detection capabilities compared to allied retinal imaging modalities (fundus photography, dye-based angiography) include collateral vessels^134^^–^^136^ and intraretinal microvascular abnormalities.^137^^–^^139^

Many of the features just described await automated or precise quantification methods based on artificial intelligence. But there are other features artificial intelligence can be used to infer and characterize that are difficult for human graders to recognize. One example is differentiating arteries and veins. Although time consuming by visual inspection, deep learning networks can accurately differentiate between these vessel types.^140^^–^^143^ Another “difficult-for-humans” task artificial intelligence can accomplish is to infer which vessels would appear leaky under dye-based angiography.^144^ This capability is useful because it could enable detection of pathological vessels without the need for an invasive procedure, and because vessel leakage obscures vasculature in dye angiography. Both these drawbacks could be avoided with an OCTA-based approach.

### Deep Learning in Diagnostics

Feature quantification is useful for disease diagnostics, prognostics, and investigations of etiology and pathophysiology. But in clinical practice we are often interested in screening, which can be done directly from an image without feature quantification using deep learning. For example, networks trained to diagnose DR directly from OCTA images have demonstrated human level performance.^145^^–^^150^ Similarly, DR-related pathology (diabetic macular ischemia) is also amenable to AI-based screening.^151^ Probably most effort has addressed DR because it is an obvious target for OCTA given the microvascular nature of the disease, but diagnostic networks exist for other prominent diseases including glaucoma^152^ and age-related macular degeneration.^153^ Because having one disease doesn't mean you can't have another, diagnosing multiple diseases simultaneously can be an efficient use of resources.^153^

Unfortunately, there is a relatively small number of publications incorporating OCTA for diagnostics; many networks get by with just structural OCT data.^154^^–^^156^ Given that many retinal diseases include a vascular component, this may be an oversight. For example, even in the case of a conventionally structural pathology (DME), OCTA data can offer quantitative advantage.^157^ Collection of larger OCTA datasets is probably the easiest way to address this limitation.

**Figure 10.**
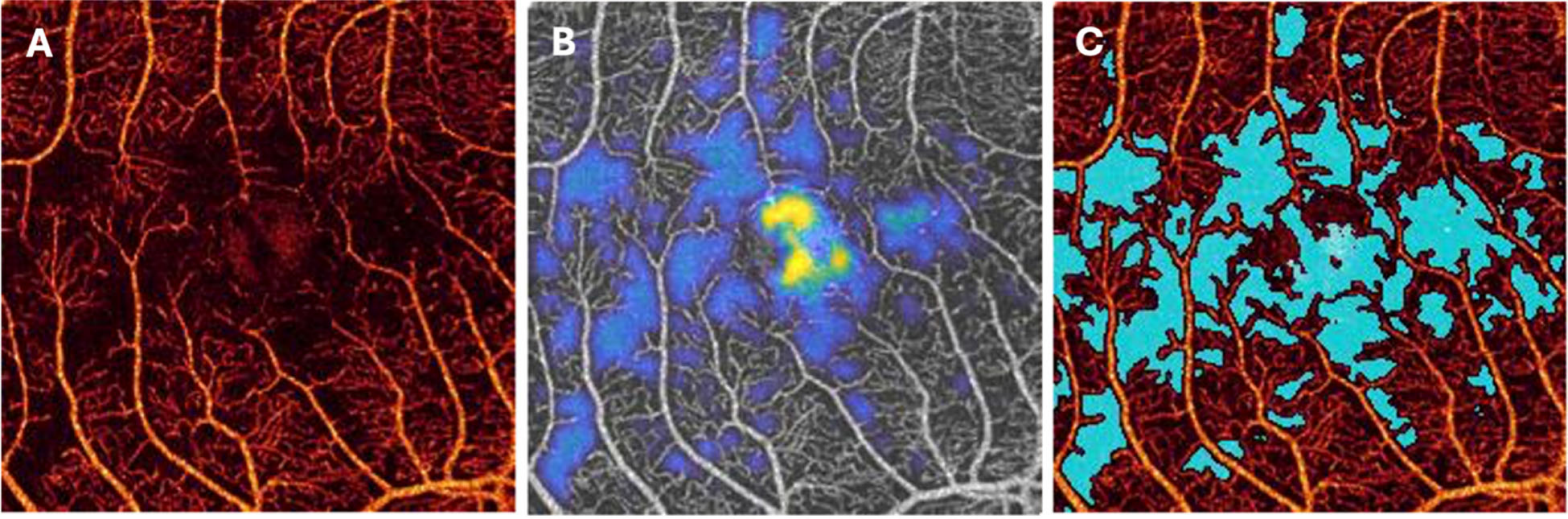
An explainability approach, biomarker activation maps (BAMs), selectively highlights clinically relevant information. Here, an example is shown for a correctly predicted referable DR scan. (**A**) Raw OCTA angiogram of the superficial vascular plexus. (**B**) Explainability framework output, showing pixels the network considered pathologic (with pathologic severity increasing from *blue* to *yellow*). (**C**) An NPA segmentation network output showing NPA in *teal*. Note the correspondence between the explainability frameworks highlighted pixels and the distribution of NPA.

An important issue for any diagnostic network is explainability. To the best of our knowledge this has not been explored in an OCTA context very thoroughly. However, explainability approaches that do incorporate OCTA information can help interpret of AI-based output (Fig. 10).^158^

## Future Directions

Perhaps the most important trends in OCTA technology can be regarded as improved acquisition speed and improved image analysis. For the latter, artificial intelligence–based approaches are playing a leading role. An important example is retinal layer segmentation, which is often the bottleneck in clinical OCTA throughput. Accurate layer segmentation is also critical for OCTA quantification, where context sensitivity for pathologies like retinal fluid is a major advantage for AI-based approaches. But these approaches rely on large datasets from which to train models. Large public datasets are not yet available for the largest fields of view, high-resolution images (in particular of deeper tissue layers), or for more specialized applications like images measuring flow speed. As these datasets are developed more potent image analysis can also be achieved. For the former (instrumentation), improved acquisition speeds opened the way to new types of measurements (flow speed) and detection sensitivity and dynamic range. And finer resolution and larger fields of view mean we can measure vascular anatomy more extensively. Because of these trends we speculate that OCTA will be increasingly used as a clinical and research technology.

## Summary

Conventional limitations to OCTA technology such as field of view, flow detection dynamic range, and imaging artifacts can now be elided. We can expect instruments to continue to improve as hardware does, and image analysis techniques to improve as more powerful machine learning algorithms are introduced.
